# Editorial: Methods and Applications of Computational Immunology

**DOI:** 10.3389/fimmu.2019.02818

**Published:** 2019-11-29

**Authors:** Benny Chain, Victor Greiff, Johannes Textor, Gur Yaari

**Affiliations:** ^1^Division of Infection and Immunity, Department of Computer Science, University College London, London, United Kingdom; ^2^Department of Immunology, University of Oslo, Oslo, Norway; ^3^Department of Tumor Immunology, Radboud Institute for Molecular Life Sciences, Nijmegen, Netherlands; ^4^Faculty of Engineering, Bar-Ilan University, Ramat Gan, Israel

**Keywords:** systems immunology, computational biology, bioinformatics, mathematical modeling, innate and adaptive immune response

Understanding the immune system is of paramount importance for the prevention and treatment of disease as well as the development of novel immunotherapies and immunodiagnostics in the framework of precision immunology and medicine. Recently, the advent of high-throughput biological methods has provided unprecedented insight into the molecular mechanisms underlying immune cell dynamics. The immense complexity of innate and adaptive immunity spanning several orders of spatial and temporal scales may, however, only be grasped by a systems computational immunology approach—specifically, by developing powerful computational approaches, which process, model, and integrate these big immunological data.

This Research Topic was designed to give a comprehensive overview of current methods and applications of computational immunology for the dissection of mammalian immunity. Twenty-nine articles are included in this Research Topic, and are categorized into the following types: 13 *Original Research* (Chaara et al.; Davidsen and Matsen; Davydov et al.; Dowling et al.; Egorov et al.; Eliyahu et al.; Gadala-Maria et al.; Meyer-Hermann et al.; Neve-Oz et al.; Priel et al.; Simon et al.; Zhou et al.; Toledano et al.), 5 *Methods* (Cohen et al.; Ma et al.; Manavalan et al.; Nouri and Kleinstein; Safonova and Pevzner), 5 *Technology Reports* (Avram et al.; Bukhari et al.; Mahajan et al.; Rosenfeld et al.; Vander Heiden et al.), 4 *Reviews* (Collins and Watson; Gfeller and Bassani-Sternberg; Schramm and Douek; Yermanos et al.), 1 *Hypothesis and Theory* (Cohen and Efroni), and 1 *Perspective* (Jansen et al.).

These papers address a broad range of conceptual challenges in computational immunology. The majority of papers focus on the development and application of computational tools for immune repertoire analysis. Specifically, they elucidate B-cell receptor phylogenetics and somatic hypermutation (Davidsen and Matsen; Schramm and Douek; Yermanos et al.), study the inference of immunoglobulin germline genes and polymorphisms (Gadala-Maria et al.; Safonova and Pevzner), shed light on immunoglobulin light chain characteristics (Collins and Watson; Toledano et al.), compare immune repertoires in aging and disease (Egorov et al.), and improve and/or develop novel computational tools for clustering immune receptor sequences (Priel et al.; Nouri and Kleinstein), immune repertoire benchmarking and error correction (Chaara et al.; Ma et al.). Furthermore, storage and standardization of immune receptor data were advanced by the development of a webserver for immunoglobulin analysis pipelines (Avram et al.), a new database of epitope-specific B-cell and T-cell receptors (Mahajan et al.), and guidelines for immune receptor data format standardization (Bukhari et al.; Vander Heiden et al.). The antigen targets of immune receptor repertoires were investigated in works on B-cell epitope prediction (Manavalan et al.) and antigen presentation (Gfeller and Bassani-Sternberg).

In addition to immune receptor biology, the dynamics of immune cells were explored for germinal center B cells (Meyer-Hermann et al.), plasma cell ontogeny (Zhou et al.), and regulatory T-cell proliferation (Dowling et al.). Immune cell signaling was investigated for cytokines (Cohen et al.), the immune synapse (Neve-Oz et al.), and macrophage function (Jansen et al.).

Finally, a conceptual paper summarized the similarities between the mammalian immune system and supervised machine learning (Cohen and Efroni).

We would like to express our deepest gratitude and appreciation to all the authors who contributed papers, and to the reviewers and editors without whose invaluable work the publication of this Research Topic would not have been possible.

## Author Contributions

All authors listed have made a substantial, direct and intellectual contribution to the work, and approved it for publication.

### Conflict of Interest

The authors declare that the research was conducted in the absence of any commercial or financial relationships that could be construed as a potential conflict of interest.

